# Assessment of the grey zone: a comparison of two methods in heart failure patients awaiting cardiac resynchronization therapy

**DOI:** 10.1186/1532-429X-13-S1-P258

**Published:** 2011-02-02

**Authors:** Simon G Duckett, Peter Koken, Anoop K Shetty, Christian Stehning, Reza Razavi, Tobias Schaeffter, Andrea J Wiethoff

**Affiliations:** 1 Kings College London, London, UK; 2 Philips Research, Hamburg, Germany

## Purpose

The purpose of this study was to compare two sequences for the assessment of the grey zone in heart failure patients.

## Background

ICD implantation is known to improve prognosis in patients with ischemic cardiomyopathy (ICM). Determining which patients are at high risk of ventricular arrhythmias and sudden death remains difficult. The presence and extent of grey zone detected by CMR has been linked to ventricular arrhythmias and may be useful in determining which patients should have an ICD.

Several processing methods to define the area of grey zone based on traditional late enhancement sequences (LGE) have been developed. Recently, a novel delayed enhancement sequence (MCDE) combined with an automated segmentation method was introduced to more accurately assess the peri-infarct area. ^1,2^ One limitation of this technique is the presence of cardiac motion in the image making the analysis cumbersome as the images from the same point in the cardiac cycle need to be filtered out of the dataset during postprocessing. An alternative option for the quantitative T1 measurement of the grey zone is the modified look-locker (MOLLI), which is not affected by cardiac motion.^3^ In the current study, the MCDE and MOLLI sequences were compared for the evaluation of the grey zone.

## Methods

Five male patients (69 ± 9yrs) with known ICM underwent CMR on a 1.5T MR-scanner (Philips Healthcare, Best, Netherlands). Approximately 15-20 minutes after injection of 0.2 mmol/kg gadobenate dimeglumine, standard LGE imaging was performed followed by two sequences to assess the grey zone covering the infarct area. The MCDE and MOLLI sequences were adapted from (1) and (3), respectively. Analysis was performed using T1-mapping software (Philips, Hamburg, Germany).

## Results

**Figure 1:**
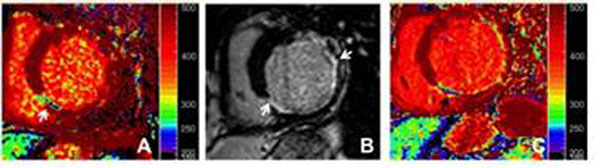
MCDE (A), LGE (B) and MOLLI (C) of patient with ICM. Area of infarct and grey zone is most closely correlated between B & C (arrows).

Both the MCDE and the MOLLI resulted in the images similar to traditional LGE with the MOLLI more closely correlated due to cardiac motion correction (Figure 1). The T1 values of both sequences fall in the range of previously published studies.

## Conclusion

The MOLLI provides an advantage over other sequences for the detection of grey zone due to cardiac motion compensation during acquisition. Accurate assessment of the grey zone has the potential to help predict which patients with ICM are at high risk of cardiac arrhythmias and therefore more likely to benefit from an ICD.
